# Effect of temperature on the pathogenesis, accumulation of viral and satellite RNAs and on plant proteome in peanut stunt virus and satellite RNA-infected plants

**DOI:** 10.3389/fpls.2015.00903

**Published:** 2015-10-29

**Authors:** Aleksandra Obrępalska-Stęplowska, Jenny Renaut, Sebastien Planchon, Arnika Przybylska, Przemysław Wieczorek, Jakub Barylski, Peter Palukaitis

**Affiliations:** ^1^Interdepartmental Laboratory of Molecular Biology, Institute of Plant Protection – National Research InstitutePoznań, Poland; ^2^Department Environmental Research and Innovation, Integrative Biology Facility, Luxembourg Institute of Science and TechnologyBelvaux, Luxembourg; ^3^Department of Molecular Virology, Adam Mickiewicz UniversityPoznań, Poland; ^4^Department of Horticultural Sciences, Seoul Women UniversitySeoul, South Korea

**Keywords:** plant-virus interactions, temperature change, plant proteomics, DIGE, cucumovirus, satellite RNA, leaf proteome, plant defense

## Abstract

Temperature is an important environmental factor influencing plant development in natural and diseased conditions. The growth rate of plants grown at C27°C is more rapid than for plants grown at 21°C. Thus, temperature affects the rate of pathogenesis progression in individual plants. We have analyzed the effect of temperature conditions (either 21°C or 27°C during the day) on the accumulation rate of the virus and satellite RNA (satRNA) in *Nicotiana benthamiana* plants infected by peanut stunt virus (PSV) with and without its satRNA, at four time points. In addition, we extracted proteins from PSV and PSV plus satRNA-infected plants harvested at 21 dpi, when disease symptoms began to appear on plants grown at 21°C and were well developed on those grown at 27°C, to assess the proteome profile in infected plants compared to mock-inoculated plants grown at these two temperatures, using 2D-gel electrophoresis and mass spectrometry approaches. The accumulation rate of the viral RNAs and satRNA was more rapid at 27°C at the beginning of the infection and then rapidly decreased in PSV-infected plants. At 21 dpi, PSV and satRNA accumulation was higher at 21°C and had a tendency to increase further. In all studied plants grown at 27°C, we observed a significant drop in the identified proteins participating in photosynthesis and carbohydrate metabolism at the proteome level, in comparison to plants maintained at 21°C. On the other hand, the proteins involved in protein metabolic processes were all more abundant in plants grown at 27°C. This was especially evident when PSV-infected plants were analyzed, where increase in abundance of proteins involved in protein synthesis, degradation, and folding was revealed. In mock-inoculated and PSV-infected plants we found an increase in abundance of the majority of stress-related differently-regulated proteins and those associated with protein metabolism. In contrast, in PSV plus satRNA-infected plants the shift in the temperature barely increased the level of stress-related proteins.

## Introduction

Temperature is one of the most important factors shaping the nature of plant pathogen interactions, as well as the normal development of healthy plants. Future changes in environmental conditions (Li et al., 2013), with an emphasis on the ambient temperature, will impact plants, plant pathogens, and consequently plant diseases (Suzuki et al., 2014; Ashoub et al., 2015). Environmental factors affect the growth, yield and populations of microorganisms living on plants, as well as vector populations, impacting the spread of plant pathogens. In the face of climate change it is of particular importance to investigate the plant performance comparisons at various temperature conditions, both when they are grown uninfected vs. challenged by various external biotic stimuli, including by important pathogens strictly dependent on the host cell life cycle, such as viruses. In the literature, there are contradictory data on the temperatures favoring viral replication, which depend on the tested virus species as well as the analyzed plant host. Higher temperatures resulted in more rigorous replication of turnip crinkle virus (TCV) in Arabidopsis plants (Zhang et al., 2012), and facilitated the spread of tobacco mosaic virus or turnip mosaic virus by weakening the plant defense responses (Király et al., 2008; Prasch and Sonnewald, 2013). On the other hand, plants infected by viruses and grown at higher temperature were less symptomatic due to more efficient RNA silencing-mediated plant defense (Szittya et al., 2003; Chellappan et al., 2005; Tuttle et al., 2008). The importance of temperature on virus spread and plant defenses, including RNA silencing, is under intense investigation (Zhong et al., 2013; Ghoshal and Sanfaçon, 2014; Patil and Fauquet, 2015). Therefore, to assess how higher temperatures might alter plant-virus interactions, we analyzed the disease progress, viral and satellite RNA (satRNA) accumulation and characterization of plant proteome differences at two temperature conditions in the model plant *Nicotiana benthamiana*, using peanut stunt virus (PSV) and its satRNA.

PSV together with *Tomato aspermy virus* (TAV) and *Cucumber mosaic virus* (CMV) are all members of the genus *Cucumovirus* in the family *Bromiviridae*. Although the host range of PSV is more restricted than that of CMV, it occurs worldwide and infects a number of important crop species, as well as the model species *N. benthamiana*. PSV contains a positive-sense (+), single-stranded (ss) RNA genome and consists of three genomic and two subgenomic strands. RNA1 and RNA2 encode components of the viral replicase complex. RNA2 additionally encodes the 2b protein that is known to participate in viral movement and suppression of RNA silencing (Netsu et al., 2008). The 2b protein is synthesized from subgenomic RNA4A (Ding et al., 1994). RNA3 contains open reading frames for the movement protein (3a) and coat protein (CP; 3b). The latter is synthesized from subgenomic RNA4 (Schwinghamer and Symons, 1975). As with some CMV strains, some PSV strains may be associated with satRNA. One of the best known examples is the PSV-P strain (Pospieszny, 1987), later sequenced and classified in subgroup I of PSV (Obrępalska-Stęplowska et al., 2008a,b). SatRNAs of PSV are linear, ssRNA molecules varying in length between 391 and 393 nt and do not encode any functional proteins (Obrępalska-Stęplowska et al., 2008a, 2012).

Infection by viruses may result in physiological changes in plants, manifested by macroscopically visible symptoms. At the molecular level, virus recognition by the host defense machinery results in the induction of plant defense responses, including those based on RNA silencing as well as others involving stress-response protein accumulation and their actions, to limit the invading pathogen accumulation (Lu et al., 2012; Pacheco et al., 2012; Zvereva and Pooggin, 2012; Obrępalska-Stęplowska et al., 2013; Fang et al., 2015). The presence of satRNA adds further to this complexity modifying both symptom expression and plant responses. Some satRNAs associated with specific strains of cucumoviruses have been proposed to either interfere with the expression or function of viral RNA silencing suppressors (Hou et al., 2011; Shen et al., 2015), or compromise the induction or accumulation of other plant stress-related proteins (Hou et al., 2011; Obrępalska-Stęplowska et al., 2013; Shen et al., 2015).

Since the levels of virus accumulation in plants are a product of actions undertaken by the host and the virus fitness under ambient conditions, we analyzed both the plant reaction at the proteome level and the levels of accumulation of virus genomic RNAs and satRNA in a susceptible host grown under both ambient and higher temperature conditions. Although the *N. benthamiana* transcriptome has been described (Nakasugi et al., 2013), many proteins are yet to be functionally characterized.

## Materials and methods

### Plants and virus materials

*N. benthamiana* plants were grown in strictly controlled conditions in separate breeding chambers with a 14 h light/10 h dark cycle, and after virus inoculations they were transferred to the appropriate temperature conditions: either 21°C day/16°C night, or 27°C day/24°C night. Infectious RNAs of PSV-P strain alone or in combination with satRNA-P (obtained as described in Obrępalska-Stęplowska et al., 2013), were used for the inoculations to *N. benthamiana* plants at the four-leaf stage, in 0.05 M phosphate buffer, pH 7.5, after dusting the leaves with Carborundum. Mock-inoculated plants (treated only with inoculation buffer) were grown as negative control at both temperatures. The plants were harvested at 21 days post inoculation (dpi), when the first symptoms of infection developed in plants maintained at 21°C.

### Comparative assessment of the accumulation of viral and satellite RNAs with real-time PCR

A comparison of the accumulation of viral genomic RNA and a subgenomic RNA, as well as satRNA, in the infected plants was done using reverse-transcription (RT) real-time (quantitative) PCR (RT-qPCR) as described previously (Obrępalska-Stęplowska et al., 2013). The RNA accumulation level was analyzed for each studied condition (mock-, PSV-, PSV and satRNA-inoculated plants) separately for five biological replicates. Each biological replicate was repeated thrice (technical replicates). The reaction was done with primers pairs hybridizing to genes encoding each of the PSV open reading-frames (Table 1) in the Corbett Rotor-Gene 6000 (Qiagen) machine. For normalization, the level of the *N. benthamiana* β-actin gene transcription was used as a reference with primers NbActA/NbAct2 (Obrępalska-Stęplowska et al., 2013). An analysis and interpretation of the results was carried out using software integrated with the Corbett machine and REST 2009 Relative Expression Software Tool V2.0.13 (Qiagen) based on the ΔΔ*Ct* method (Pfaffl et al., 2002).

**Table 1:** **Primers used in reverse-transcription quantitative PCR analysis of the changes in the PSV and satellite RNA accumulation**.

**Primer name**	**Sequence**	**Region of amplification**
PSVq1F	5′ CTTCTGCCCTCGTTGATAAAG 3′	PSV OFR1a
PSVq1R	5′ CATACCGATTTCGAATCACTTC 3′	
PSVq2aF	5′ CTTCTAGGTATCCCCGTAAG 3′	PSV ORF2a
PSVq2aR	5′ CAAGCACATTGATACCCTATC 3′	
PSVq2bF	5′ CTCmTATCCTCCCAGCTAyAC 3′	PSV ORF2b
PSVq2bR	5′ GAATAACTrCCCTCACACCAC 3′	
PSVq3aF	5′ CTAGTCGGACTTTAACACAAC 3′	PSV ORF3a
PSVq3aR	5′ ACGCTCATATATCCCTTAGAC 3′	
PSVqCPF	5′ ACACATACACTTCGTTGGATG 3′	PSV ORFCP
PSVqCPR	5′ CCTTCwTCTTCGGAAATTCAG 3′	
PARNA1	5′ GGGAGGGCGGGCGTTCGTAGTG 3′	satRNA
PARNA2	5′ GCCGTGGCCTTTCGTGGTC 3′	

### Accumulation of viral RNAs

Accumulation of PSV RNAs and satRNA was assessed by means of RT-qPCR. For this purpose whole plants infected with the virus were taken individually and were pulverized in liquid nitrogen using a mortar and pestle. A sample of the ground material was used for total RNA extraction using Trizol solution (Life Technologies. The RNA accumulation levels were analyzed for each studied condition separately for five biological replicates. Each biological replicate was repeated thrice (technical replicates). Aliquots of 0.5–1.0 μg total RNA were first treated with RNase-free DNase and subsequently used for cDNA synthesis using Maxima H Minus First Strand cDNA Synthesis Kit supplemented with DNase (Life Technologies). The samples with 1.0 μg of starting total RNA were diluted using an equal volume of water. Accumulation of RNA1, RNA3, and satRNA were done by RT-qPCR using the Power SYBR® Green PCR Master Mix (Applied Biosystems), the corresponding primers (Table 1) hybridizing to regions encoding for 1a, and CP proteins, or the satRNA, according to manufacturer's protocol. The reactions were performed with 9 μl of master mix and 1 μl of the cDNA (ca. 25 ng of used template RNA). For each set of primers a standard curve was prepared from a series of 10-fold dilutions of respective templates: for viral and satellite RNAs infectious clones of PSV were used (Obrępalska-Stęplowska et al., 2013). The relative distribution of the RNAs was normalized using *N. benthamiana* β-actin mRNA.

### Protein extraction and labeling

After 21 dpi, plants were harvested from each treatment (PSV-P, PSV-P/satRNA, and mock-inoculated plants, grown at both 21°C and 27°C). Four plants of similar growth were chosen for proteomic analyses. Fresh leaf materials (300 mg) from each plant were ground in liquid nitrogen and proteins were extracted and labeled as previously described (Obrępalska-Stęplowska et al., 2013). Samples containing 30 μg of each Cy2 labeled internal standard, plus Cy3 and Cy5 labeled proteins for comparisons were pooled and lysis buffer (7 M urea, 2 M thiourea, 0.5% CHAPS) was added to a total volume of 120 μl. Per the sampling conditions, four replicates were used (two labeled with Cy3 and two with Cy5). The samples representing biological replicates were tested separately.

### 2D-DIGE and image capture and analysis

The following steps were done essentially as described previously (Obrępalska-Stęplowska et al., 2013). Briefly, 24 cm IPG strips, pH 3–10 NL (Bio-Rad) were rehydrated overnight with the 450 μl of rehydration solution [the mixture of the Destreak rehydration solution (GE Healthcare) and Bio-lyte (Bio-Rad) per strip]. Proteins were cup-loaded on IPG strips and IEF was performed on an Ettan IPG-phor Manifold (GE Healthcare) with the following settings: initially, for 3 h at 150 V, then up to 1000 V over 5 h, a constant 1000 V for 4 h, then up to 10,000 V over 10 h and a final step at 10,000 V to reach 80,000 V-hr. Afterwards, strips containing proteins were equilibrated for 15 min in Equilibration buffer (Gel Company, San Francisco, CA) with an addition of 36% (w/v) urea and 1% (w/v) DTT. The strips then were equilibrated for another 15 min using the Equilibration buffer with 36% (w/v) urea and 2.5% (w/v) IAA, after which the strips were sealed with low-melt agarose (Gel Company) on top of the precast 12.5% acrylamide/bisacrylamide gels (Gel Company). Separation of proteins was done in an Ettan Dalt 12 apparatus (GE Healthcare) at 2.5 W/gel and at 15°C. After electrophoresis, gels with proteins labeled with Cy2, Cy3, and Cy5 were scanned with a Typhoon Variable Mode Imager 9400 (GE Healthcare). The collected images were analyzed using the Decyder v7.0 software (GE Healthcare). Spots were detected, and after matching and comparisons spot of interest, these were taken for mass spectrometry analysis.

### Protein identification

Picking, digestion with trypsin and spotting on MALDI targets were done as described by Renaut et al. (2006), with trypsin concentration of 8 μg/ml. Then each sample (0.7 μl) was deposited onto the target analyzed on a 4800 MALDI-TOF/TOF instrument (ABSciex, Foster City, CA, USA). Subsequently, searches were carried out using Mascot software in the NCBI “*Viridiplantae*” database (downloaded on 23 Sep 2013, 32770904 sequences), the “*viruses*” database (1220058 sequences), as well as EST for *Nicotiana* databases (downloaded on 2nd July 2014, 7936392 sequences). The search parameters allowed carboxyamidomethylation of cysteine (as fixed modification) and as oxidation of methionine and tryptophan and pyro-glu for N-term E or N-term Q (as variable modifications). A mass window of 100 ppm for peptide mass tolerance and 0.5 Da for the fragment mass tolerance were allowed in searches. Homology identification was retained with a probability set at 95% and where needed, the identifications were verified manually.

### Statistical analysis

For a statistical analyses of the proteomic data, an ANOVA One-way and a *post-hoc* Student's *t*-test One-way ANOVA were applied and a significance level lower than *p* < 0.05 was taken into account. Statistically valid average changes >1.3 and < -1.3 between treatments were considered.

### *In silico* analysis of protein functions

Since annotation of many identified proteins and ESTs turned out to be incomplete we performed an array of complementary analyses to determine their functions. First, we applied Blast2GO software (Conesa and Götz, 2008) to associate GO terms with Mascot hits. This tool utilizes information on top BLAST hits (we performed local BLASTp or BLASTx search with *e*-value cut-off of 1e-25 against set of all protein sequences of *Solanaceae* and *Arabidopsis thaliana* from RefSeq database) and predicted protein domains to annotate the query. Additionally we used KAAS (KEGG Automatic Annotation Server, Moriya et al., 2007) to acquire KEGG annotations. This tool classifies each sequence to an orthologous cluster and retrieves annotations assigned to it. Finally, we performed direct BLAST (Altschul et al., 1997) search against the tomato proteome (*Solanum lycopersicum*, up000004994) and manually gathered annotation from publicly available databases in order to compare identified proteins with sequences from well-annotated relatives. All obtained information were carefully curated and compared. KAAS identifications were used to project expression data onto KEGG metabolic networks of Tomato using CyKEGGParser (Cytoscape plugin developed by Nersisyan et al., 2014).

## Results

In this study, our aim was to assess changes occurring in plants that were grown at 27°C, when sister plants grown at 21°C started developing the first symptoms of virus expression, with regard to virus accumulation and specific plant responses. Additionally, prior to inoculation, all of the plants were grown at the same temperature (24°C) and were inoculated at a similar stage of growth. Some plants were inoculated with transcripts of genomic RNAs of PSV-P with or without satRNA, while others were inoculated with purified viral particles of wild-type PSV-P, naturally containing satRNA. In all cases, the symptoms were monitored. The first set of inoculated plants was used for proteomic analysis of the impact of the temperature on mock-inoculated, PSV-infected, and PSV plus satRNA-infected plants simultaneously, together with the relative comparison of viral and satRNA accumulation under these conditions. The second set of inoculated plants was used for the measurement of viral RNA and satRNA levels at various time points after inoculation.

### Growth and symptoms appearance

Plants maintained under different temperature conditions grew at different rates. Faster growth and leaf development were observed under the higher temperature conditions (27°C). Likewise, the progress of symptom development also was observed to be more rapid for plants kept at 27°C than at 21°C. In the former case, the onset of symptom development was observed about 14 dpi, while in the latter case about 21 dpi. Moreover, more rapid symptom development was observed in plants inoculated with purified viral particles vs. those plants inoculated with infectious genomic transcripts of PSV.

### Comparison of viral RNA and satRNA levels in plants grown under different temperatures

**Figure 1:**
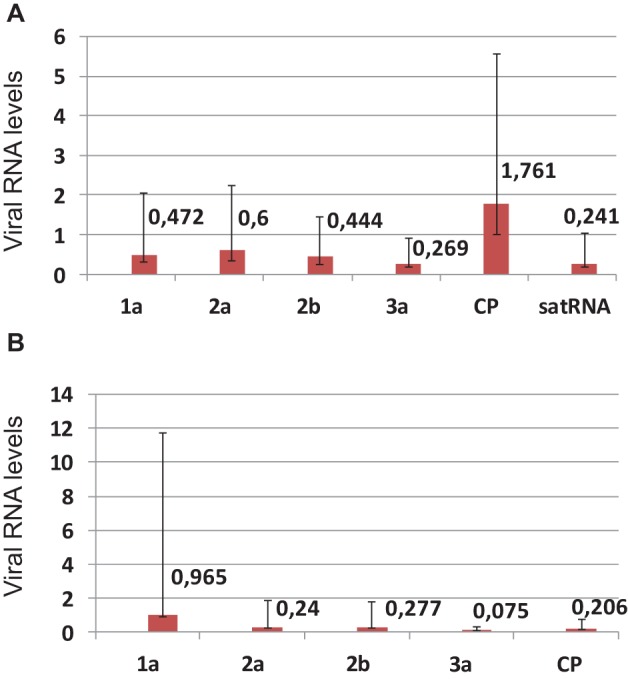
**Real-time (quantitative) PCR (RT-qPCR) analysis of the changes in the levels of PSV RNAs and satRNA in *Nicotiana benthamiana* plants infected with biologically infectious transcripts of PSV-P plus satRNA (A), or PSV RNAs alone (B) at 21 dpi, comparing differences in accumulation between plants maintained at 27°C vs. 21°C**. In **(A)**, the change in 2a-encoding RNA was statistically insignificant and in the **(B)** the change in 1a-endoding RNA was statistically insignificant. The error bars represent standard errors.

A comparison was made of the accumulation levels of genomic and subgenomic RNAs of the virus with or without satRNA, at 21 dpi. In general, a decrease in accumulation of the analyzed RNAs was observed at higher temperature vs. the lower temperature (Figures 1A,B), with the exception of RNA4, the subgenomic RNA expressing the CP ORF, when plants were infected by PSV plus satRNA (Figure 1A). That the exception is in RNA4 accumulation rather than RNA3 and RNA4, was shown by the reduced accumulation of the RNA3, containing the 3a ORF (Figure 1A). Similarly, a four-fold drop in satRNA accumulation was observed in plants maintained at the higher temperature (Figure 1A).

### Accumulation of PSV RNA and satRNA during a time course of infection at two temperatures

**Figure 2:**
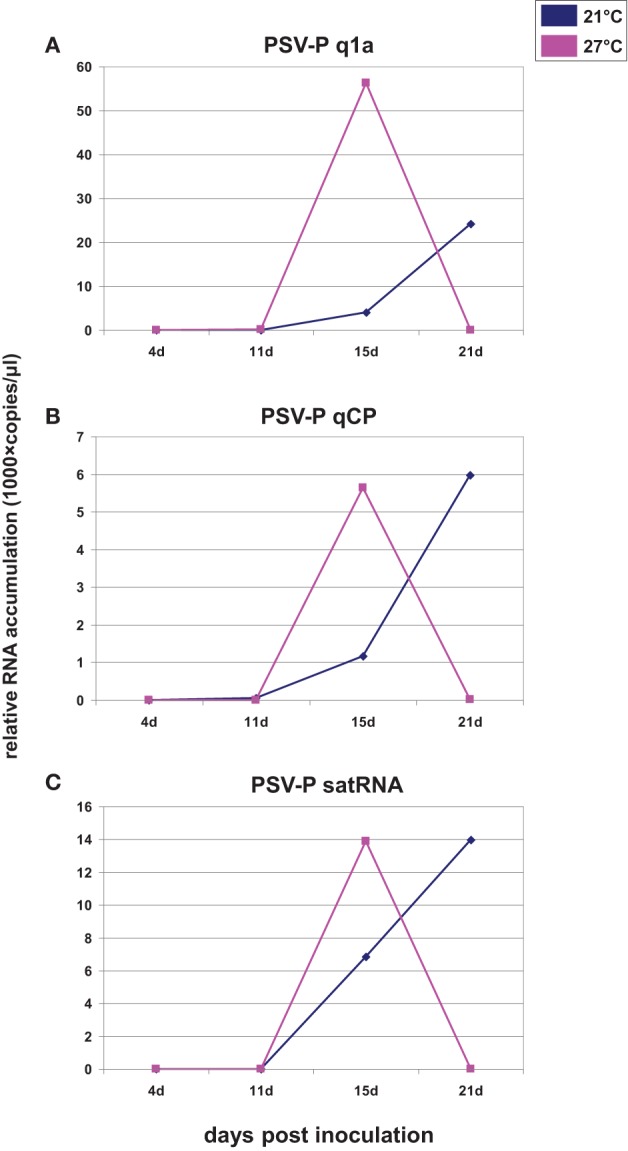
**Quantification of the number of copies of ORF1 (A), ORF3b (CP; B), and satRNA (C) assessed on the basis of accumulation of all relative to reference gene**. The inoculum was wild-type PSV-P naturally containing satRNA. X axis shows the time points analyzed and Y axis shows the number of copies (in thousands) of analyzed RNAs. At 21 dpi, the level of ORF1a, CP, and satRNA tended toward zero. The experiment was repeated twice with similar results. The results derived from a single experiment are shown.

Since most of published data showed that viral RNA accumulation was greater at higher temperatures, (e.g., Király et al., 2008), we wanted to determine if plants infected with PSV and maintained at a higher temperature were accumulating viral RNAs less efficiently than those maintained at a lower temperature, or if PSV-infected plants reached a plateau of viral RNA accumulation earlier if grown at a higher temperature, followed by a drop in accumulation levels. In the latter case, this would suggest that at 21 dpi we were observing a reduced accumulation at the higher temperature while the plants grown at the lower temperature were showing a gradual increase. Thus, we conducted a time course of viral RNA accumulation over four time points in the infected plants, by RT-qPCR at the two temperatures, using primers specific to the RNAs containing ORF1 (RNA1) and ORF3b (CP; RNA3 and RNA4; Figures 2A,B) and to the satRNA (Figure 2C). The analyses showed that over 21 days of observation the accumulation levels of viral and satRNAs at the higher temperature increased rapidly after 11 dpi and then decreased between 15 and 21 dpi. On the other hand, in plants grown at the lower temperature, viral RNA accumulation increased gradually after 11 dpi, which then accelerated between 15 and 21 dpi. Thus, at 21 dpi the amounts of accumulated RNAs 1–3 and subgenomic RNA4, as well as satRNA, were all much lower at 27°C than at 21°C (Figure 2). The data show the accumulation profiles for viral genes and the satRNA. The differences in the scales may be due to primer performance. Importantly, the symptom expression associated with the high accumulation of virus started to develop just before 15 dpi in plants grown at 27°C and about 21 dpi in plants grown at 21°C (not shown).

### Proteome comparison of PSV infected (with or without satRNA) and mock-inoculated plants grown at two temperatures

To characterize the effect of plant maintenance at different temperatures on its proteome, *N. benthamiana* plants were grown over the same time period in separate greenhouse chambers with temperature sets at 21°C or 27°C during the day. When the first symptoms of infection were visible on the plants grown at 21°C, leaves of all plants were harvested. At this time point, plants grown at 27°C all had fully developed symptoms of disease and were significantly taller than those grown at 21°C. All plants were first tested for the presence or absence of PSV RNA and satRNA, as appropriate, and then used for proteomic profiling using DIGE analysis followed by Maldi TOF/TOF mass spectrometry and Mascot searches.

The plants were analyzed in three separate sets comparing the proteome analysis results for the two temperatures: (1) between mock-inoculated plants; (2) between plants infected by transcripts of the genomic PSV RNAs; and (3) between plants infected by both transcripts of the genomic PSV plus satRNA. On the basis of DIGE analysis and MS/Mascot searches 38, 57, and 28 differentially-accumulated protein spots were found in mock-inoculated, PSV-infected, and PSV plus satRNA-infected plants, respectively, among which 23, 42, and 24, respectively, were identified and functionally annotated (Tables 2–**4**).

**Table 2:** **Differentially-accumulated proteins in mock-inoculated *Nicotiana benthamiana***.

**Control (mock-inoculated plants)**					
**Spot**	**Mascot hit**	***T*****-test**	Av ratio^*^	**Curated name**	**Other GOs**
**PHOTOSYNTHESIS AND CARBOHYDRATE METABOLIC PROCESS**					
1412	gi|77745458|gb|ABB02628.1|	0.0035	1.82	Triosephosphate isomerase	–
810	gi|445628|prf||1909374A	0.024	1.62	Rubisco activase	Response to stimulus
1262	gi|12643758|sp|Q40565.1|RCA2_TOBAC	0.045	−1.39	Rubisco activase	Response to stimulus
963	gi|362799991|dbj|BAL41455.1|	0.047	−1.41	Fructose 1,6, bisphosphate aldolase	–
1319	gi|62865755|gb|AAY17070.1|	0.025	−1.45	Carbonic anhydrase	–
768	gi|159227386|emb|CAJ80484.1|	0.028	−1.62	ATP synthase CF1 beta subunit	–
1302	gi|3914940|sp|O20252.1|S17P_SPIOL	0.045	−1.94	Sedoheptulose-1,7-bisphosphatase, chloroplastic	–
1896	gi|190146228|gb|FG644758.1|FG644758	0.0077	−2	Photosystem II reaction center PSB28 protein	–
1295	gi|62865755|gb|AAY17070.1|	0.038	−7.27	Carbonic anhydrase	–
**CELLULAR AMINO ACID METABOLIC PROCESS**					
770	gi|12643806|sp|Q9LEC8.1|DHEB_NICPL	0.014	−1.63	Glutamate dehydrogenase	Response to stimulus
**PROTEIN METABOLIC PROCESS**					
1540	gi|357480975|ref|XP_003610773.1|	0.029	2.83	Proteasome subunit beta type-2-A-like	–
1407	gi|14594931|emb|CAC43326.1|	0.044	2.46	Proteasome subunit beta type-5-like	–
1741	gi|460407957|ref|XP_004249415.1|	0.048	1.86	Ubiquitin-conjugating enzyme E2 variant 1D	Response to stimulus
1814	gi|508712318|gb|EOY04215.1|	0.0089	1.66	Ribosomal protein PSRP3/Ycf65	–
678	gi|39857235|gb|CK284052.1|CK284052	0.0034	1.52	Peptidase m20 m25 m40 family protein	–
**RESPONSE TO STIMULUS**					
1555	gi|134642|sp|P22302.1|SODF_NICPL	0.026	2.99	Superoxide dismutase [Fe]	–
1059	gi|460385950|ref|XP_004238663.1|	0.0024	1.97	Quinone oxidoreductase-like protein	Photosynthesis
1601	gi|543176812|gb|AGV54429.1|	0.014	1.8	Peroxiredoxin	Photosynthesis
1194	gi|460397188|ref|XP_004244150.1|	0.035	−1.48	Thiamine thiazole synthase 1, chloroplastic-like	–
**TRANSPORT**					
405	gi|350537279|ref|NP_001234287.1|	0.015	−1.69	Vacuolar H+-ATPase A2 subunit	–
406	gi|350537279|ref|NP_001234287.1|	0.014	−1.8	Vacuolar H+-ATPase A2 subunit	–
408	gi|350537279|ref|NP_001234287.1|	0.013	−1.86	Vacuolar H+-ATPase A2 subunit	–
**NO ANNOTATION AVAILABLE**					
1101	gi|52580767|gb|CV292958.1|CV292958	0.031	2.12	Oxidoreductase, zinc binding dehydrogenase family protein	–

*Av. ratio = the average ratio derived from four measurements. Av. ratio cells are colored according to the value (red represents highest, green lowest and yellow intermediate ratios). T-test column is colored according to p-values (lowest values are green, higher ones are paler)*.

### Influence of temperature on plant proteome in mock-inoculated plants

In the case of mock-inoculated plants, most of the differentially-accumulated proteins were identified as being involved in photosynthesis and processes associated with general carbohydrate metabolism (11; seven decreased in abundance at the higher temperature), protein metabolic processes (five; all increased), or response to stimulus (eight; five increased) (Table 2). Also, a decrease was observed in the amounts of three protein spots for vacuolar H+-ATPase A2 subunit potentially involved in intra and intercellular transport (Table 2).

### Influence of temperature on the plant proteome in PSV-infected plants

When plants infected with PSV were compared at two temperatures, the majority of the identified differentially-accumulated proteins were involved in carbohydrate metabolic processes including photosynthesis (Table 3). These were present in smaller quantities in comparison to plants grown at the lower temperature. Only one protein (involved in glycolysis) was more abundant. Similarly, as for mock-inoculated plants, the proteins involved in protein metabolic processes mostly increased in accumulation (11 of 14; Table 3). However, also among the identified differentially-accumulated proteins are those participating in the processes of synthesis, assembly (including chaperones and chaperone-like) and degradation. Twenty differentially-accumulating protein spots were identified as involved in plant responses to stimulus. Among these, 12 proteins were more abundant and eight proteins were less abundant. Most proteins identified within this group could function in other processes, such as: five in photosynthesis (all less abundant); two for methionine synthase in cellular amino acid metabolic processes (both more abundant); and five in protein metabolic processes [ubiquitin-conjugating enzyme E2, heat shock 70 kDa protein (2 spots), chaperonin CPN60, and peptidyl-prolyl cis-trans isomerase; all more abundant at 27°C]. Three other proteins from the last category were less abundant, including spots containing hsp70 and peptidyl-prolyl cis-trans-isomerase. Several proteins in this group showed both an increase in abundance as well as a decrease in abundance, in different spots (Table 3). These include heat shock proteins and peptidyl-prolyl cis-trans isomerase. This might reflect the reaction of various forms or isoforms of these proteins to the applied conditions, as well as the fact that they have a role in both protein metabolism and plant responses to external stimulus. Interestingly, two proteins (spots no 1119 and 1929) involved in RNA interactions also were observed in higher amounts (Table 3); the first one might also participate in plant reactions to external stresses. This suggests there may be the intense re-organization within infected cells.

**Table 3:** **Differentially-accumulated proteins in *Nicotiana benthamiana* infected by PSV**.

**Peanut stunt virus*****—*****infected plants**					
**Spot**	**Mascot hit**	***T*****-test**	**Av ratio**	**Curated name**	**Other GOs**
**PHOTOSYNTHESIS AND CARBOHYDRATE METABOLIC PROCESS**					
1412	gi|77745458|gb|ABB02628.1|	0.0079	1.55	Triosephosphate isomerase	–
1908	gi|548526|sp|P35477.1|PLAS2_TOBAC	0.038	−1.33	Plastocyanin	–
939	gi|460400830|ref|XP_004245935.1|	0.047	−1.53	Phosphoribulokinase, chloroplastic-like	–
1302	gi|3914940|sp|O20252.1|S17P_SPIOL	0.043	−1.68	Sedoheptulose-1,7-bisphosphatase, chloroplastic	–
620	gi|460411525|ref|XP_004251160.1|	0.016	−1.69	Rubisco large subunit-binding protein subunit alpha (chaperonin 60 alpha)	Response to stimulus
1129	gi|460373374|ref|XP_004232495.1|	0.036	−1.81	Ferredoxin–NADP reductase, leaf isozyme, chloroplastic-like	Response to stimulus
1050	gi|356624532|pdb|3T15|A	0.027	−1.86	Rubisco activase	Response to stimulus
1490	gi|4930130|pdb|1RPX|A	0.045	−2.06	Ribulose-phosphate 3-epimerase	–
1113	gi|350536711|ref|NP_001234005.1|	0.013	−2.16	Glyoxisomal malate dehydrogenase	–
686	gi|460401720|ref|XP_004246365.1|	0.045	−2.2	Fructose-1,6-bisphosphatase, chloroplastic-like	Photosynthesis
706	gi|460401720|ref|XP_004246365.1|	0.0079	−2.23	Fructose-1,6-bisphosphatase, chloroplastic-like	Photosynthesis
1294	gi|508726009|gb|EOY17906.1|	0.023	−2.23	Chloroplast sedoheptulose-1,7-bisphosphatase	Response to stimulus
1319	gi|62865755|gb|AAY17070.1|	0.03	−2.38	Carbonic anhydrase	–
696	gi|7708648|emb|CAB89998.1|	0.0064	−2.41	ATP synthase CF1 beta subunit	–
802	gi|460370413|ref|XP_004231047.1|	0.024	−2.57	Glycerate dehydrogenase (hydroxypyruvate reductase), peroxisomal	–
874	gi|362799991|dbj|BAL41455.1|	0.013	−3.1	Fructose 1,6, bisphosphate aldolase	–
1084	gi|460373374|ref|XP_004232495.1|	0.0014	−3.61	Ferredoxin–NADP reductase, leaf isozyme, chloroplastic-like	Response to stimulus
1030	gi|255566555|ref|XP_002524262.1|	0.024	−3.98	Malate dehydrogenase	–
**CELLULAR AMINO ACID METABOLIC PROCESS**					
1425	gi|76870188|gb|DV161180.1|DV161180	0.0085	2.59	Arginine biosynthesis bifunctional protein ArgJ	–
256	gi|8439545|gb|AAF74983.1|AF082893_1	0.0034	2.07	Methionine synthase	Response to stimulus
237	gi|530704703|gb|AGT40326.1|	0.039	1.95	Methionine synthase	Response to stimulus
797	gi|190813280|gb|FG200828.1|FG200828	0.016	−1.41	Aminomethyltransferase, mitochondrial (Glycine cleavage system T protein)	–
**PROTEIN METABOLIC PROCESS**					
1540	gi|357480975|ref|XP_003610773.1|	0.038	2.63	Proteasome subunit beta type-2-A-like	–
1867	gi|311124976|gb|HS084903.1|HS084903	0.008	2.33	FKBP-type peptidyl-prolyl cis-trans isomerase	–
1828	gi|28261753|ref|NP_783267.1|	0.042	2.14	Ribosomal protein L14	–
477	gi|255554262|ref|XP_002518171.1|	0.0033	1.85	Chaperonin CPN60	Response to stimulus
1845	gi|94330105|gb|EB683707.1|EB683707	0.0058	1.84	FKBP-type peptidyl-prolyl cis-trans isomerase	–
1655	gi|356536583|ref|XP_003536816.1|	0.0028	1.65	Peptidyl-prolyl cis-trans isomerase	Response to stimulus
1754	gi|460407957|ref|XP_004249415.1|	0.018	1.55	Ubiquitin-conjugating enzyme E2 variant 1D	Response to stimulus
678	gi|39857235|gb|CK284052.1|CK284052	0.01	1.41	Peptidase m20 m25 m40 family protein	–
1096	gi|460407825|ref|XP_004249350.1|	0.047	1.37	Cochaperone GrpE family protein	–
620	gi|460411525|ref|XP_004251160.1|	0.016	−1.69	Rubisco large subunit-binding protein subunit alpha (chaperonin 60 alpha)	Response to stimulus
1657	gi|460365069|ref|XP_004228428.1|	0.015	−3.43	Peptidyl-prolyl cis-trans isomerase	Response to stimulus
**RESPONSE TO STIMULUS**					
388	gi|460369188|ref|XP_004230445.1|	0.0029	2.73	Heat shock 70 kda protein	Protein metabolic process, transport
1809	gi|134616|sp|P27082.2|SODC_NICPL	0.031	1.94	Superoxide dismutase [Cu-Zn]	–
1204	gi|460373807|ref|XP_004232705.1|	0.03	1.75	Lactoylglutathione lyase-like (glyoxalase homolog)	–
424	gi|460369188|ref|XP_004230445.1|	0.028	1.66	Heat shock 70 kda protein	Protein metabolic process, transport
1236	gi|39859660|gb|CK285269.1|CK285269	0.0023	1.63	NADH-cytochrome b5 reductase-like protein-like	–
852	gi|304368145|gb|ADM26718.1|	0.029	−2.22	Glycolate oxidase	–
328	gi|460369188|ref|XP_004230445.1|	0.01	−2.35	Heat shock 70 kda protein	Protein metabolic process, transport
**RNA BINDING**					
1119	gi|460391607|ref|XP_004241412.1|	0.038	1.69	Stem-loop (RNA) binding protein of 41 kda chloroplastic-like	Response to stimulus
1929	gi|47512378|gb|CN747381.1|CN747381	0.018	1.56	Ribonucleoprotein	–
**TRANSPORT**					
1940	gi|254629991|gb|FS376425.1|FS376425	0.022	1.72	Nuclear transport factor 2-like	Response to stimulus

*Av. ratio cells are colored according to the value (red represents highest, green lowest and yellow intermediate ratios). T-test column is colored according to p-values (lowest values are green, higher ones are paler)*.

### Influence of temperature on the plant proteome in PSV-infected plants in the presence of satRNA

A comparison of plants grown under two temperature conditions and co-infected with PSV and its satRNA showed less abundant accumulation of proteins associated with photosynthesis and carbohydrate metabolism at higher temperature than was observed at the lower temperature (Table 4). There was a greater reduction in the amount of these proteins in PSV plus satRNA-infected plants, when compared to the plants infected with PSV alone, grown under the corresponding conditions. This decrease is especially evident for such proteins as ribulose bisphosphate carboxylase/oxygenase (RuBisCo) small subunit, RuBisCo large subunit and carbonic anhydrase (Table 4). Plant defense was less pronounced and only seven protein spots classified as being involved in plant response to stimulus were identified, four decreasing in abundance at the higher temperature and three increasing (Table 4); only chaperonin 20, peptidyl-prolyl cis-trans isomerase (both in protein metabolic processes), and peroxiredoxin (associated with photosynthetic activities) were more abundant. One of two proteins identified as involved in nucleotide binding was more abundant, while the second was less abundant. The rest of the identified proteins associated with small molecule metabolic processes and transport were observed as less abundant at the higher temperature of growth (Table 4).

**Table 4:** **Differentially-accumulated proteins in *Nicotiana benthamiana* infected by PSV plus satRNA**.

***Peanut stunt virus*** **plus satRNA-co-infected plants**					
**Spot**	**Mascot hit**	***T*****-test**	**Av ratio**	**Curated name**	**Other GOs**
**PHOTOSYNTHESIS AND CARBOHYDRATE METABOLIC PROCESS**					
338	gi|37359708|dbj|BAC98299.1|	0.045	−1.61	Beta-xylosidase alpha-l-arabinofuranosidase	–
336	gi|37359708|dbj|BAC98299.1|	0.029	−1.49	Beta-xylosidase alpha-l-arabinofuranosidase	–
365	gi|37359708|dbj|BAC98299.1|	0.0053	−1.49	Beta-xylosidase alpha-l-arabinofuranosidase	–
363	gi|254658228|gb|FS388360.1|FS388360	0.00049	−1.55	Beta-d-xylosidase 7-like	–
1864	gi|3914596|sp|Q39748.1|RBS6_FLAPR	0.018	−2.5	Ribulose-1,5-bisphosphate carboxylase, small subunit precursor	–
1365	gi|22550386|gb|AAL51055.2|AF454759_1	0.029	−2.72	Carbonic anhydrase	–
1361	gi|62865755|gb|AAY17070.1|	0.042	−2.93	Carbonic anhydrase	–
1877	gi|13241101|gb|AAK16227.1|AF044395_1	0.049	−3.14	Ribulose-1,5-bisphosphate carboxylase small chain 3b	–
1344	gi|115473|sp|P27141.1|CAHC_TOBAC	0.026	−3.29	Carbonic anhydrase	–
546	gi|1352794|sp|P48709.1|RBL_NICDE	0.042	−3.57	Ribulose-1,5-bisphosphate carboxylase/oxygenase large subunit	–
1303	gi|62865755|gb|AAY17070.1|	0.036	−4.24	Carbonic anhydrase	–
1379	gi|62865755|gb|AAY17070.1|	0.042	−4.83	Carbonic anhydrase	–
1868	gi|225905973|gb|ACO35888.1|	0.047	−4.96	Ribulose-1,5-bisphosphate carboxylase, small subunit	–
1885	gi|47512534|gb|CN747537.1|CN747537	0.0076	−7.04	Ribulose bisphosphate carboxylase small chain, chloroplastic	Response to stimulus
1362	gi|62865755|gb|AAY17070.1|	0.036	−7.12	Carbonic anhydrase	–
**CELLULAR AMINO ACID METABOLIC PROCESS**					
832	gi|40457328|gb|AAR86719.1|	0.045	−2.13	Glutamine synthetase	–
**PROTEIN METABOLIC PROCESS**					
1489	gi|15242045|ref|NP_197572.1|	0.039	1.64	Chaperonin 20	Response to stimulus
1655	gi|356536583|ref|XP_003536816.1|	0.015	1.59	Peptidyl-prolyl cis-trans isomerase	Response to stimulus
**RESPONSE TO STIMULUS**					
1601	gi|543176812|gb|AGV54429.1|	0.028	1.52	Peroxiredoxin	Photosynthesis
523	gi|117689576|gb|DB688765.1|DB688765	0.019	−1.54	Glutathione reductase	–
1752	gi|460365514|ref|XP_004228646.1|	0.044	−1.57	Zkt protein containing k-box and a tpr region	–
**RNA BINDING**					
1910	gi|94326102|gb|EB679704.1|EB679704	0.017	2.44	RNA recognition motif-containing protein	–
1346	gi|133248|sp|P19683.1|ROC4_NICSY	0.042	−1.82	31 kDa RNA binding protein	Response to stimulus
**TRANSPORT**					
406	gi|350537279|ref|NP_001234287.1|	0.034	−1.5	Vacuolar H+-ATPase A2 subunit	–

*Av. ratio cells are colored according to the value (red represents highest, green lowest and yellow intermediate ratios). T-test column is colored according to p-values (lowest values are green, higher ones are paler)*.

### Comparison of all treatment conditions

**Figure 3:**
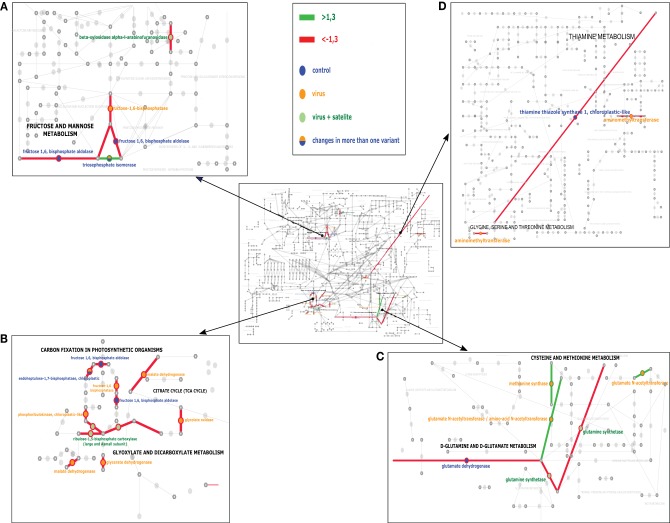
**The KEGG analysis of the pathways most affected at the experimental temperature for control (mock-inoculated) plants—blue, PSV-infected plants—yellow, and PSV plus satRNA-infected plants—green circles**. Green and red lines indicate the pathways joined by the proteins which were found to be more abundant or less abundant (respectively). The middle panel shows the general view of the studied pathways. **(A,B)** focus on carbohydrate metabolism and **(C,D)** focus on amino acid metabolism.

To establish the general impact of the temperature on the plants grown under the different infection conditions, we compared all the results to identify the proteins that in all treatments were either less abundant or more abundant (Table 5). In the present study, changes in the levels of proteins taking part in the Calvin cycle, tricarboxylic acid (TCA) cycle, glycolysis, as well as fructose and mannose metabolism were observed (Figure 3). Overall, a differential abundance of 11 protein spots in carbohydrate metabolism (including photosynthesis) was observed for mock-inoculated plants at the two temperature conditions, among which the levels of seven decreased at the higher temperature. In the case of spots for RuBisCo activase-containing sequences, both increased and decreased accumulation were found, which might be associated with its function in the maintenance of CO_2_ fixation (Law and Crafts-Brandner, 2001) and in increasing the rate of photosynthesis during temperature changes (Yamori et al., 2012). In the case of virus-challenged plants, 18 protein spots involved in photosynthesis and carbohydrate metabolism differentially accumulated and 17 of these were in lesser amounts at the higher temperature; not only proteins involved in photosynthesis, but also proteins in the TCA cycle were found in smaller quantities during high temperature stress (Gammulla et al., 2011). Infection by PSV plus satRNA caused a decrease of 15 out of 16 differentially-accumulating proteins involved in photosynthesis and carbohydrate metabolism. This shows clearly that within the temperature range examined the increase in temperature caused a decrease in the level of proteins with a role in carbohydrate metabolism, which was particularly obvious in plants infected by PSV and PSV plus satRNA.

**Table 5:** **Data summary of proteins that are more abundant (green) or less abundant (pink) at higher temperature under all conditions**.

**No**.	**Curated name**	**Av. Ratio (mock-inoculation)**	**Av. Ratio (PSV-inoculation)**	**Av. Ratio(PSV + satRNA-inoculation)**	**Processess**
363	Beta-d-xylosidase 7-like	−1.74	−1.24	−**1.55**	Photosynthesis and carbohydrate metabolism
377	Malate dehydrogenase	−1.61	−1.61	−**1.42**	Photosynthesis and carbohydrate metabolism
696	ATP synthase beta subunit	−1.46	−**2.41**	−1.46	Photosynthesis and carbohydrate metabolism
874	Fructose-bisphosphate aldolase	−1.58	−**3.1**	−1.9	Photosynthesis and carbohydrate metabolism
1294	Sedoheptulose-1; 7-bisphosphatase	−2.09	−**2.23**	−2.15	Photosynthesis carbohydrate metabolism/ response to stimulus
1295	Carbonic anhydrase	−**7.27**	−5.38	−1.56	Photosynthesis and carbohydrate metabolism
1302	Sedoheptulose-1; 7-bisphosphatase; chloroplastic	−**1.94**	−**1.68**	−2.28	Photosynthesis and carbohydrate metabolism
1303	Carbonic anhydrase	−3.06	−7.05	−**4.24**	Photosynthesis and carbohydrate metabolism
1344	Carbonic anhydrase	−2.1	−3	−**3.29**	Photosynthesis and carbohydrate metabolism
1361	Carbonic anhydrase	−2.19	−3.42	−**2.93**	Photosynthesis and carbohydrate metabolism
1362	Carbonic anhydrase	−3.95	−15.34	−**7.12**	Photosynthesis and carbohydrate metabolism
1365	Carbonic anhydrase	−2.56	−4.19	−**2.72**	Photosynthesis and carbohydrate metabolism
1379	Carbonic anhydrase	−2.8	−14.32	−**4.83**	Photosynthesis and carbohydrate metabolism
1657	Peptidyl-prolyl cis-trans isomerase	−1.57	−**3.43**	−1.52	Protein metabolism/response to stimuli
1686	23 kDa polypeptide of photosystem II oxygen-evolving complex	−**1.84**	−1.47	−1.48	Photosynthesis
1896	Photosystphotosystem II reaction center PSB28 protein	−**2**	−1.32	−1.42	Photosynthesis and carbohydrate metabolism
1885	Ribulose bisphosphate carboxylase small chain, chloroplastic	−1.25	−2.05	−**7.04**	Photosynthesis and carbohydrate metabolism/response to stimuli
523	Glutathione reductase	−1.35	−1.84	−**1.54**	Response to stimuli
405	Vacuolar H+-ATPase A2 subunit	−**1.69**	−1.37	−1.58	Transport
406	Vacuolar H+-ATPase A2 subunit	−**1.8**	−1.38	−**1.5**	Transport
408	Vacuolar H+-ATPase A2 subunit	−**1.86**	−1.27	−1.37	Transport
1059	Quinone oxidoreductase-like protein	**1.97**	1.24	1.21	Response to stimuli/ photosynthesis and carbohydrate metabolism
1119	Stem-loop (RNA) binding protein of 41 kda chloroplastic-like	1.49	**1.69**	1.31	Response to stimuli/ RNA binding
1910	Ribonucleoprotein; chloroplast; putative	1.36	1.49	**2.44**	RNA binding
1555	Superoxide dismutase [Fe]	**2.99**	1.75	1.21	Response to stimuli
1601	Peroxiredoxin	**1.8**	1.21	**1.52**	Response to stimuli/ photosynthesis
1655	Peptidyl-prolyl cis-trans isomerase	1.34	**1.65**	**1.59**	Response to stimuli/ protein metabolism
1845	Peptidyl-prolyl cis-trans isomerase FKBP12-like	1.37	**1.84**	1.45	Response to stimuli/ protein metabolism
1867	FKBP-type peptidyl-prolyl cis-trans isomerase	1.43	**2.33**	1.5	Response to stimuli/ protein metabolism
1540	Proteasome subunit beta type-2-A-like	**2.83**	**2.63**	1.31	Protein metabolism
1425	Arginine biosynthesis bifunctional protein ArgJ	1.32	**2.59**	1.28	Cellular amino acid metabolic process

*The average ratio (marked Av ratio) that is statistically significant is marked in bold*.

It is also interesting to note the changes in the proteins involved in protein and amino acid metabolism in plants maintained at the two temperatures. Mock-inoculated plants grown at the higher temperature all had five differentially-accumulated identified spots that were more abundant. However, four of them, were mostly involved in protein degradation (proteasome subunit beta type-2 and 5, ubiquitin-conjugating enzyme E2 and peptidase m20/m25/m40 family), and one protein was in the plastid ribosome function (ribosomal protein PSRP3). On the other hand, plants infected by PSV have 14 differentially-accumulated protein spots due to the change of temperature. Among these, 11 are more abundant at the higher temperature. These include proteins involved in processes of synthesis, degradation and transformation, including chaperones, likewise found previously (Gammulla et al., 2011). This indicates an intense effect on protein metabolism in virus-infected plants.

There are only a few differentially-accumulating proteins for which the fold-change for the two tested conditions is statistically significant (Table 5). Nevertheless, from this comparison it is evident that proteins involved in photosynthesis, carbohydrate metabolism and transport all decreased in accumulation, while proteins involved in plant defense and some aspects of protein metabolism increased in accumulation.

No statistically significant decrease or increase of abundance of either the pathogenesis responsive proteins, or proteins involved in RNA silencing was observed in all three comparisons, which might result from either there being no change in the levels of these proteins under the studied conditions, or these proteins levels being below the limit of detection.

### Annotation and visualization of the expression data

Blast2GO assigned provisional names and GO terms to 83 (100%) of the Mascot hits. Direct BLAST search against the tomato proteome revealed 69 putative homologs and allowed us to annotate 36 of them with at least one KEGG term. The KAAS server, on the other hand, mapped only 57 proteins to 40 orthologous clusters (which suggests a degree of redundancy in the analyzed set), but assigned 37 of them to KEGG pathways. Results of the final curation and data comparison are presented in Supplementary Table S1, while proposed functional classification is shown in Tables 2–4. Only a part of the differently expressed proteins could be successfully mapped on Tomato KEGG pathways. While some of them could not be assigned to orthologous clusters, other failed to map despite successful assignment. This suggests that KEGG ontology for *S. lycopersicum* may still be incomplete and some clusters are missing. Results of the mapping on selected pathways are shown in Figure 3 and indicate the carbohydrate and amino acid metabolic pathways were influenced the most by the temperature factor under the three conditions studied: PSV infection, PSV plus satRNA infection, and growth without virus challenge.

## Discussion

In plants infected by PSV or PSV plus satRNA, the maximum level of symptoms development was reached earlier for the plants grown at the higher temperature, as observed also for *N. benthamiana* plants infected by the geminiviruses African cassava mosaic virus (ACMV) and Sri Lancan cassava mosaic virus (Chellappan et al., 2005). However, in that study, the severity of symptoms was greater for plants grown at the lower temperature. Moreover, those authors observed that at higher temperatures newly emerged leaves of the ACMV-infected plants showed recovery from symptom appearance, while this was not the case in our study. In our system, on the day the plants were harvested (21 dpi), the accumulation of viral RNAs and satRNA was higher at the lower temperature, as was confirmed by RT-qPCR, with the exception of the subgenomic RNA encoding the CP, which was higher at 27°C when PSV plus satRNA-infected plants were analyzed. [This is consistent with proteomic data for this condition where larger amounts of CP were found at 27°C than at 21°C; however, differences in PSV CP accumulation in the proteomic part were statistically not significant (not shown).] A similar situation was observed also in the study by Chellappan et al. (2005), where the virus genomic DNA levels were higher at the lower temperature than at the higher temperature studied, for both geminiviruses. Those authors suggested that the temperature played a major role in decreasing the virus load.

As expected, at the higher temperature, better growth of the plants was observed. The onset of symptom development and their severity were associated with the level of the virus in host cells and clearly suggested major changes in plants metabolism. There are a number of reports on the effect of external abiotic stimuli on plant development, photosynthetic performance and other aspects of primary metabolism (Eastburn et al., 2011; Kosová et al., 2011; Obrępalska-Stęplowska et al., 2013; Wituszyñska et al., 2013; Rodziewicz et al., 2014; Farrant and Ruelland, 2015; George et al., 2015). In the present study, the changes caused by virus infection and temperature could be separated. In addition, the temperature range used was within that supporting the normal growth and not associated with heat/cold stress. Other studies have examined the effects of infection by pathogenic factors resulting in increases of various elements of the defense machinery including pathogenesis-related proteins (Elvira et al., 2008), heat-shock proteins (Lu et al., 2003; Chen et al., 2008), and RNA silencing (Vance and Vaucheret, 2001; Waterhouse et al., 2001). Zhang et al. (2012) tested other temperature sets for their analysis of interactions of TCV in Arabidopsis showing that higher temperature (26°C) facilitated more rigorous replication of this virus than was in the case in plants grown at a lower temperature (18°C). Our results are consistent with this observation.

When mock-inoculated plants grown at the two temperature conditions were compared, the levels of proteins in the categories “protein metabolic process” and “response to stimulus” increased moderately at the higher temperature. The former group represented mostly factors participating in the degradation processes. The increase in peroxiredoxin levels, with a role in oxidative stress control, also was found in another study (Mühlhaus et al., 2011). Proteins in other categories largely decreased moderately. Altogether, it appears that at higher temperature basal plant responses to external stimuli performed better than at 21°C.

The presence of virus is generally known to diminish accumulation of photosynthetic proteins in infected plants (Di Carli et al., 2010; Obrępalska-Stęplowska et al., 2013; Wu et al., 2013; Fang et al., 2015). In this study, this effect was even more intensified in virally-infected plants that were maintained at the higher temperature. This might be the result of not only an increase in temperature but also from an advanced stage of viral infection. Plants respond by production of heat shock proteins to various stresses, and these proteins constitute part of the plant's adaptive mechanisms for which increased accumulation was observed both at high and low temperature stresses (Timperio et al., 2008). Overall, the results show clearly that the plant response to external factors is more active at the higher temperature, especially when challenged by the virus.

The temperature-dependent induction of plant resistance response to viral infection also was observed previously (Malamy et al., 1992; Baulcombe and Dean, 2014; Prasch and Sonnewald, 2015). The reduced levels of a few proteins might result from their primary role; e.g., in photosynthesis, which seems to perform worse at higher temperature (see below). An increase of proteins functioning in RNA binding was observed and might reflect the reaction to infection by an RNA pathogen (Li and Nagy, 2011). The effectiveness of the plant response was observed among others in Arabidopsis challenged by a bacterial pathogen, together with increased level of symptom expression. In that case this plant reaction was thought to be associated with host-pathogen interaction, rather than just an increase in pathogen growth at the higher temperature (Eastburn et al., 2011), which might be also the case in the present study.

SatRNA slightly delayed symptom expression on plants infected with PSV-P at 21°C (Obrępalska-Stęplowska et al., 2013). The presence satRNA in the PSV infection at the higher temperature resulted in fewer differentially-accumulated proteins than for PSV-infected plants, and either did not influence plant defense proteins, or decreased their level of expression (Obrępalska-Stęplowska et al., 2013).

The most affected proteins in the three studied conditions were those participating in photosynthesis and carbohydrate metabolism. The changes in photosynthetic protein abundance in plants challenged by pathogens is a known phenomenon (Sajnani et al., 2007; Garavaglia et al., 2010; Eastburn et al., 2011; Obrępalska-Stęplowska et al., 2013), as this process is among those more sensitive to environmental stressors. Usually, photosynthesis is repressed in response to various types of pathogens (Roberts and Paul, 2006; Major et al., 2010; Kangasjärvi et al., 2012). In mock-inoculated plants and PSV-infected, proteins involved in protein degradation increased in abundance at the higher temperature. Similar responses of proteins responsible for protein degradation also were found in rice leaves exposed to changing temperature (Gammulla et al., 2011). The intense effect on protein metabolism in virus-infected plants can be explained by the necessity to ensure synthesis of various cell compounds, as well as plant-derived metabolites for growth to maintain the cellular integrity of host cells, which is important during early phases of infection by biotrophic and hemibiotrophic pathogens (Kangasjärvi et al., 2012). It might also be of importance for the phase of restoration of metabolism in later stages of infection. Importantly, cellular amino acid metabolic processes also were affected. Most of proteins with this role were higher in abundance. This might be associated with high demands on the host during virus propagation and with cellular recovery of the host after the viral infection has been suppressed. This is significantly less evident in cells infected together by PSV and its satRNA where only one protein spot differentially accumulating at the two temperature conditions was identified. Peptidyl-prolyl-cis trans isomerase is known not only to participate in protein metabolism but also in response to various abiotic stresses including those associated with temperature (Marivet et al., 1994; Gammulla et al., 2011). It seems that in this respect protein metabolism is comparable for plants maintained at the two temperature conditions.

Overall, when all proteomic data for the three examined situations (PSV-infection, PSV plus satRNA-infection and no virus treatment) were compared, it is conceivable that at the higher temperature transport was affected, although not all the differences in the data were statistically significant.

## Conclusions

Summarizing all the data, higher temperature caused a faster growth rate, disease symptom development on plants and initial accumulation of virus, which after reaching a plateau, dropped precipitously. Plants grown at the higher temperature showed a reduction in accumulation of proteins involved in photosynthesis and carbohydrate metabolism as well as transport, and an increase in accumulation of proteins involved in plant response to external stimuli, as well as some involved in protein metabolism. This is especially evident in *N. benthamiana* infected by PSV, where a decrease of proteins involved in the TCA cycle was observed, and to a lesser extent in mock-inoculated and PSV plus satRNA-infected plants. Additionally, an increase in abundance of proteins involved in amino acid metabolism was observed for PSV-infected plants grown at higher temperature.

### Conflict of interest statement

The authors declare that the research was conducted in the absence of any commercial or financial relationships that could be construed as a potential conflict of interest.

## Supplementary material

Supplementary Table S1 **Collective characteristics of identified proteins including their Mascot, Blast2GO, KEGG, and KAAS ortholog definition as well as and KEGG pathway names classified on the basis of *Solanum lycopersicum* database**.

## References

[B1] Altschul S. F. Madden T. L. Schäffer A. A. Zhang J. Zhang Z. Miller W.. (1997). Gapped BLAST and PSI-BLAST: a new generation of protein database search programs. Nucleic Acids Res. 25, 3389–3402. 10.1093/nar/25.17.3389 9254694 PMC146917

[B2] Ashoub A. Baeumlisberger M. Neupaertl M. Karas M. Brüggemann W. (2015). Characterization of common and distinctive adjustments of wild barley leaf proteome under drought acclimation, heat stress and their combination. Plant Mol. Biol. 87, 459–471. 10.1007/s11103-015-0291-4 25647426

[B3] Baulcombe D. C. Dean C. (2014). Epigenetic regulation in plant responses to the environment. Cold Spring Harb. Perspect. Biol. 6:a019471. 10.1101/cshperspect.a019471 25183832 PMC4142964

[B4] Chellappan P. Vanitharani R. Ogbe F. Fauquet C. M. (2005). Effect of temperature on geminivirus-induced RNA silencing in plants. Plant Physiol. 138, 1828–1841. 10.1104/pp.105.066563 16040661 PMC1183375

[B5] Chen Z. Zhou T. Wu X. Hong Y. Fan Z. Li H. (2008). Influence of cytoplasmic heat shock protein 70 on viral infection of *Nicotiana benthamiana*. Mol. Plant Pathol. 9, 809–817. 10.1111/j.1364-3703.2008.00505.x 19019009 PMC6640221

[B6] Conesa A. Götz S. (2008). Blast2GO: a comprehensive suite for functional analysis in plant genomics. Int. J. Plant Genomics 2008:619832. 10.1155/2008/619832 18483572 PMC2375974

[B7] Di Carli M. Villani M. E. Bianco L. Lombardi R. Perrotta G. Benvenuto E.. (2010). Proteomic analysis of the plant- virus interaction in Cucumber mosaic virus (CMV) resistant transgenic tomato. J. Proteome Res. 9, 5684–5697. 10.1021/pr100487x 20815412

[B8] Ding S.-W. Anderson B. J. Haase H. R. Symons R. H. (1994). New overlapping gene encoded by the cucumber mosaic virus genome. Virology 198, 593–601. 10.1006/viro.1994.1071 8291242

[B9] Eastburn D. McElrone A. Bilgin D. (2011). Influence of atmospheric and climatic change on plant–pathogen interactions. Plant Pathol. 60, 54–69. 10.1111/j.1365-3059.2010.02402.x

[B10] Elvira M. I. Galdeano M. M. Gilardi P. García-Luque I. Serra M. T. (2008). Proteomic analysis of pathogenesis-related proteins (PRs) induced by compatible and incompatible interactions of pepper mild mottle virus (PMMoV) in *Capsicum chinense* L3 plants. J. Exp. Bot. 59, 1253–1265. 10.1093/jxb/ern032 18375936

[B11] Fang X. Chen J. Dai L. Ma H. Zhang H. Yang J.. (2015). Proteomic dissection of plant responses to various pathogens. Proteomics 15, 1525–1543. 10.1002/pmic.201400384 25641875

[B12] Farrant J. M. Ruelland E. (2015). Plant signalling mechanisms in response to the environment. Environ. Exp. Bot. 114, 1–3. 10.1016/j.envexpbot.2015.02.006

[B13] Gammulla C. G. Pascovici D. Atwell B. J. Haynes P. A. (2011). Differential proteomic response of rice (*Oryza sativa*) leaves exposed to high−and low−temperature stress. Proteomics 11, 2839–2850. 10.1002/pmic.201100068 21695689

[B14] Garavaglia B. S. Thomas L. Gottig N. Zimaro T. Garofalo C. G. Gehring C.. (2010). Shedding light on the role of photosynthesis in pathogen colonization and host defense. Commun. Integr. Biol. 3, 382–384. 10.4161/cib.3.4.12029 20798833 PMC2928325

[B15] George I. S. Pascovici D. Mirzaei M. Haynes P. A. (2015). Quantitative proteomic analysis of cabernet sauvignon grape cells exposed to thermal stresses reveals alterations in sugar and phenylpropanoid metabolism. Proteomics 15, 3048–3060. 10.1002/pmic.201400541 25959233

[B16] Ghoshal B. Sanfaçon H. (2014). Temperature-dependent symptom recovery in *Nicotiana benthamiana* plants infected with tomato ringspot virus is associated with reduced translation of viral RNA2 and requires ARGONAUTE 1. Virology 456, 188–197. 10.1016/j.virol.2014.03.026 24889238

[B17] Hou W. N. Duan C. G. Fang R.-X. Zhou X.-Y. Guo H.-S. (2011). Satellite RNA reduces expression of the 2b suppressor protein resulting in the attenuation of symptoms caused by Cucumber mosaic virus infection. Mol. Plant Pathol. 12, 595–605. 10.1111/j.1364-3703.2010.00696.x 21722297 PMC6640352

[B18] Kangasjärvi S. Neukermans J. Li S. Aro E.-M. Noctor G. (2012). Photosynthesis, photorespiration, and light signalling in defence responses. J. Exp. Bot. 63, 1619–1636. 10.1093/jxb/err402 22282535

[B19] Király L. Hafez Y. M. Fodor J. Király Z. (2008). Suppression of tobacco mosaic virus-induced hypersensitive-type necrotization in tobacco at high temperature is associated with downregulation of NADPH oxidase and superoxide and stimulation of dehydroascorbate reductase. J. Gen. Virol. 89, 799–808. 10.1099/vir.0.83328-0 18272772

[B20] Kosová K. Vítámvás P. Prášil I. T. Renaut J. (2011). Plant proteome changes under abiotic stress—contribution of proteomics studies to understanding plant stress response. J. Proteomics 74, 1301–1322. 10.1016/j.jprot.2011.02.006 21329772

[B21] Law R. D. Crafts-Brandner S. J. (2001). High temperature stress increases the expression of wheat leaf ribulose-1, 5-bisphosphate carboxylase/oxygenase activase protein. Arch. Biochem. Biophys. 386, 261–267. 10.1006/abbi.2000.2225 11368350

[B22] Li J. Lin X. Chen A. Peterson T. Ma K. Bertzky M.. (2013). Global priority conservation areas in the face of 21st century climate change. PLoS ONE 8:e54839. 10.1371/journal.pone.0054839 23359638 PMC3554607

[B23] Li Z. Nagy P. D. (2011). Diverse roles of host RNA binding proteins in RNA virus replication. RNA Biol. 8, 305–315. 10.4161/rna.8.2.15391 21505273 PMC3230553

[B24] Lu J. Du Z.-X. Kong J. Chen L.-N. Qiu Y.-H. Li G.-F.. (2012). Transcriptome analysis of *Nicotiana tabacum* infected by Cucumber mosaic virus during systemic symptom development. PLoS ONE 7:e43447. 10.1371/journal.pone.0043447 22952684 PMC3429483

[B25] Lu R. Malcuit I. Moffett P. Ruiz M. T. Peart J. Wu A. J.. (2003). High throughput virus−induced gene silencing implicates heat shock protein 90 in plant disease resistance. EMBO J. 22, 5690–5699. 10.1093/emboj/cdg546 14592968 PMC275403

[B26] Major I. T. Nicole M.-C. Duplessis S. Séguin A. (2010). Photosynthetic and respiratory changes in leaves of poplar elicited by rust infection. Photosyn. Res. 104, 41–48. 10.1007/s11120-009-9507-2 20012201

[B27] Malamy J. Hennig J. Klessig D. F. (1992). Temperature-dependent induction of salicylic acid and its conjugates during the resistance response to tobacco mosaic virus infection. Plant Cell 4, 359–366. 10.1105/tpc.4.3.359 12297650 PMC160135

[B28] Marivet J. Margis-Pinheiro M. Frendo P. Burkard G. (1994). Bean cyclophilin gene expression during plant development and stress conditions. Plant Mol. Biol. 26, 1181–1189. 10.1007/BF00040698 7811975

[B29] Moriya Y. Itoh M. Okuda S. Yoshizawa A. C. Kanehisa M. (2007). KAAS: an automatic genome annotation and pathway reconstruction server. Nucleic Acids Res. 35, W182–W185. 10.1093/nar/gkm321 17526522 PMC1933193

[B30] Mühlhaus T. Weiss J. Hemme D. Sommer F. Schroda M. (2011). Quantitative shotgun proteomics using a uniform 15N-labeled standard to monitor proteome dynamics in time course experiments reveals new insights into the heat stress response of Chlamydomonas reinhardtii. Mol. Cell. Proteomics 10:M110004739. 10.1074/mcp.M110.004739 21610104 PMC3186191

[B31] Nakasugi K. Crowhurst R. N. Bally J. Wood C. C. Hellens R. P. Waterhouse P. M. (2013). De novo transcriptome sequence assembly and analysis of RNA silencing genes of *Nicotiana benthamiana*. PLoS ONE 8:59534. 10.1371/journal.pone.0059534 23555698 PMC3610648

[B32] Nersisyan L. Samsonyan R. Arakelyan A. (2014). CyKEGGParser: tailoring KEGG pathways to fit into systems biology analysis workflows. F1000Res. 3:145. 10.12688/f1000research.4410.2 25383185 PMC4215754

[B33] Netsu O. Hiratsuka K. Kuwata S. Hibi T. Ugaki M. Suzuki M. (2008). Peanut stunt virus 2b cistron plays a role in viral local and systemic accumulation and virulence in *Nicotiana benthamiana*. Arch. Virol. 153, 1731–1735. 10.1007/s00705-008-0166-y 18663407

[B34] Obrępalska-Stęplowska A. Budziszewska M. Pospieszny H. (2008a). Complete nucleotide sequence of a Polish strain of Peanut stunt virus (PSV-P) that is related to but not a typical member of subgroup I. Acta Biochim. Pol. 55, 731–739. 19081851

[B35] Obrępalska-Stęplowska A. Budziszewska M. Wieczorek P. Czerwoniec A. (2012). Analysis of two strains of Peanut stunt virus: satRNA-associated and satRNA free. Virus Genes 44, 513–521. 10.1007/s11262-012-0729-6 22392626

[B36] Obrępalska-Stęplowska A. Nowaczyk K. Budziszewska M. Czerwoniec A. Pospieszny H. (2008b). The sequence and model structure analysis of three Polish peanut stunt virus strains. Virus Genes 36, 221–229. 10.1007/s11262-007-0180-2 18049887

[B37] Obrępalska-Stęplowska A. Wieczorek P. Budziszewska M. Jeszke A. Renaut J. (2013). How can plant virus satellite RNAs alter the effects of plant virus infection? A study of the changes in the *Nicotiana benthamiana* proteome after infection by Peanut stunt virus in the presence or absence of its satellite RNA. Proteomics 13, 2162–2175. 10.1002/pmic.201200056 23580405

[B38] Pacheco R. García-Marcos A. Manzano A. de Lacoba M. G. Camañes G. García-Agustín P.. (2012). Comparative analysis of transcriptomic and hormonal responses to compatible and incompatible plant-virus interactions that lead to cell death. Mol. Plant Microbe Interact. 25, 709–723. 10.1094/MPMI-11-11-0305 22273391

[B39] Patil B. L. Fauquet C. M. (2015). Light intensity and temperature affect systemic spread of silencing signal in transient agroinfiltration studies. Mol. Plant Pathol. 16, 484–494. 10.1111/mpp.12205 25220764 PMC6638542

[B40] Pfaffl M. W. Horgan G. W. Dempfle L. (2002). Relative expression software tool (REST©) for group-wise comparison and statistical analysis of relative expression results in real-time PCR. Nucleic Acids Res. 30, e36–e36. 10.1093/nar/30.9.e36 11972351 PMC113859

[B41] Pospieszny H. (1987). Wirius karłowatości orzecha ziemnego (*Peanut stunt virus*)—nowo rozpoznany patogen roślin motylkowatych w Polsce. Prace Nauk. Inst. Ochr. Roślin 28, 27–85.

[B42] Prasch C. M. Sonnewald U. (2013). Simultaneous application of heat, drought, and virus to Arabidopsis plants reveals significant shifts in signaling networks. Plant Physiol. 162, 1849–1866. 10.1104/pp.113.221044 23753177 PMC3729766

[B43] Prasch C. M. Sonnewald U. (2015). Signaling events in plants: stress factors in combination change the picture. Environ. Exp. Bot. 114, 4–14. 10.1016/j.envexpbot.2014.06.020

[B44] Renaut J. Hausman J. F. Wisniewski M. E. (2006). Proteomics and low-temperature studies: bridging the gap between gene expression and metabolism. Physiol. Plant. 126, 97–109. 10.1111/j.1399-3054.2006.00617.x

[B45] Roberts M. R. Paul N. D. (2006). Seduced by the dark side: integrating molecular and ecological perspectives on the influence of light on plant defence against pests and pathogens. New Phytol. 170, 677–699. 10.1111/j.1469-8137.2006.01707.x 16684231

[B46] Rodziewicz P. Swarcewicz B. Chmielewska K. Wojakowska A. Stobiecki M. (2014). Influence of abiotic stresses on plant proteome and metabolome changes. Acta Physiol. Plant. 36, 1–19. 10.1007/s11738-013-1402-y

[B47] Sajnani C. Zurita J. L. Roncel M. Ortega J. M. Barón M. Ducruet J. M. (2007). Changes in photosynthetic metabolism induced by tobamovirus infection in *Nicotiana benthamiana* studied *in vivo* by thermoluminescence. New Phytol. 175, 120–130. 10.1111/j.1469-8137.2007.02074.x 17547672

[B48] Schwinghamer M. W. Symons R. H. (1975). Fractionation of cucumber mosaic virus RNA and its translation in a wheat embryo cell-free system. Virology 63, 252–262. 10.1016/0042-6822(75)90389-X 163049

[B49] Shen W. X. Au P. C. Shi B. J. Smith N. A. Dennis E. S. Guo H.. (2015). Satellite RNAs interfere with the function of viral RNA silencing suppressors. Front. Plant Sci. 6:281. 10.3389/fpls.2015.00281 25964791 PMC4408847

[B50] Suzuki N. Rivero R. M. Shulaev V. Blumwald E. Mittler R. (2014). Abiotic and biotic stress combinations. New Phytol. 203, 32–43. 10.1111/nph.12797 24720847

[B51] Szittya G. Silhavy D. Molnár A. Havelda Z. Lovas A. Lakatos L.. (2003). Low temperature inhibits RNA silencing-mediated defence by the control of siRNA generation. EMBO J. 22, 633–640. 10.1093/emboj/cdg74 12554663 PMC140757

[B52] Timperio A. M. Egidi M. G. Zolla L. (2008). Proteomics applied on plant abiotic stresses: role of heat shock proteins (HSP). J. Proteomics 71, 391–411. 10.1016/j.jprot.2008.07.005 18718564

[B53] Tuttle J. R. Idris A. M. Brown J. K. Haigler C. H. Robertson D. (2008). Geminivirus-mediated gene silencing from Cotton leaf crumple virus is enhanced by low temperature in cotton. Plant Physiol. 148, 41–50. 10.1104/pp.108.123869 18621976 PMC2528111

[B54] Vance V. Vaucheret H. (2001). RNA silencing in plants-defense and counterdefense. Science 292, 2277–2280. 10.1126/science.1061334 11423650

[B55] Waterhouse P. M. Wang M.-B. Lough T. (2001). Gene silencing as an adaptive defence against viruses. Nature 411, 834–842. 10.1038/35081168 11459066

[B56] Wituszyñska W. Gałązka K. Rusaczonek A. Vanderauwera S. Van Breusegem F. Karpiñski S. (2013). Multivariable environmental conditions promote photosynthetic adaptation potential in *Arabidopsis thaliana*. J. Plant Physiol. 170, 548–559. 10.1016/j.jplph.2012.11.016 23287000

[B57] Wu L. Han Z. Wang S. Wang X. Sun A. Zu X.. (2013). Comparative proteomic analysis of the plant–virus interaction in resistant and susceptible ecotypes of maize infected with sugarcane mosaic virus. J. Proteomics 89, 124–140. 10.1016/j.jprot.2013.06.005 23770298

[B58] Yamori W. Masumoto C. Fukayama H. Makino A. (2012). Rubisco activase is a key regulator of non−steady−state photosynthesis at any leaf temperature and, to a lesser extent, of steady−state photosynthesis at high temperature. Plant J. 71, 871–880. 10.1111/j.1365-313X.2012.05041.x 22563799

[B59] Zhang X. Zhang X. Singh J. Li D. Qu F. (2012). Temperature-dependent survival of Turnip crinkle virus-infected arabidopsis plants relies on an RNA silencing-based defense that requires dcl2, AGO2, and HEN1. J. Virol. 86, 6847–6854. 10.1128/JVI.00497-12 22496240 PMC3393596

[B60] Zhong S.-H. Liu J.-Z. Jin H. Lin L. Li Q. Chen Y.. (2013). Warm temperatures induce transgenerational epigenetic release of RNA silencing by inhibiting siRNA biogenesis in Arabidopsis. Proc. Natl. Acad. Sci. U.S.A. 110, 9171–9176. 10.1073/pnas.1219655110 23686579 PMC3670308

[B61] Zvereva A. S. Pooggin M. M. (2012). Silencing and innate immunity in plant defense against viral and non-viral pathogens. Viruses 4, 2578–2597. 10.3390/v4112578 23202495 PMC3509663

