# Corrigendum: Effect of temperature on the pathogenesis, accumulation of viral and satellite RNAs and on plant proteome in peanut stunt virus and satellite RNA-infected plants

**DOI:** 10.3389/fpls.2016.00839

**Published:** 2016-06-07

**Authors:** Aleksandra Obrępalska-Stęplowska, Jenny Renaut, Sebastien Planchon, Arnika Przybylska, Przemysław Wieczorek, Jakub Barylski, Peter Palukaitis

**Affiliations:** ^1^Interdepartmental Laboratory of Molecular Biology, Institute of Plant Protection—National Research InstitutePoznań, Poland; ^2^Department of Environmental Research and Innovation, Integrative Biology Facility, Luxembourg Institute of Science and TechnologyBelvaux, Luxembourg; ^3^Department of Molecular Virology, Adam Mickiewicz UniversityPoznań, Poland; ^4^Department of Horticultural Sciences, Seoul Women UniversitySeoul, South Korea

**Keywords:** plant-virus interactions, temperature change, plant proteomics, DIGE, cucumovirus, satellite RNA, leaf proteome, plant defense

Reason for Corrigendum:

There was a mistake in the grant number in acknowledgment section as published. The correct version appears below. The authors apologize for the mistake. This error does not change the scientific conclusions of the article in any way.

This paper was supported by the Polish National Center of Science grant UMO-2011/03/B/NZ9/01577 to AOS. PP is funded by grant no. NRF-2013R1A2A2A01016282 from the Korean National Research Foundation of the Republic of Korea.

## Author contributions

All authors listed, have made substantial, direct and intellectual contribution to the work, and approved it for publication.

## Funding

This paper was supported by the Polish National Center of Science grant UMO-2011/03/B/NZ9/01577 to AOS. PP is funded by grant no. NRF-2013R1A2A2A01016282 from the Korean National Research Foundation of the Republic of Korea.

### Conflict of interest statement

The authors declare that the research was conducted in the absence of any commercial or financial relationships that could be construed as a potential conflict of interest.

